# *In-vitro* Investigation of Polyphenol-Rich Date (*Phoenix dactylifera L*.) Seed Extract Bioactivity

**DOI:** 10.3389/fnut.2021.667514

**Published:** 2021-08-23

**Authors:** Serene Hilary, Jaleel Kizhakkayil, Usama Souka, Fatima Al-Meqbaali, Wissam Ibrahim, Carine Platat

**Affiliations:** Department of Nutrition and Health, College of Medicine and Health Sciences, United Arab Emirates University, Al Ain, United Arab Emirates

**Keywords:** date seeds, polyphenols, anti-hyperglycemic effect, antioxidant, anti-adipogenic effect, PPARγ, GLUT4

## Abstract

Date seeds are a by-product of the date fruit processing industry with minimal human use; however, they are a rich source of polyphenols with a range of potential biological properties. The current study investigates the cytotoxicity of date seed polyphenols against cancer cell lines, its ability to combat hyperglycemia, its antioxidant potential and its anti-adipogenic effect. The present work aimed to establish the usefulness of date seeds in the food industry as a functional ingredient. The anti-tumour activity of DSE was tested in a panel of cell lines such as MCF-7, MDA-MB-231, Hep-G2, Caco-2, and PC-3 by measuring cell viability and cleaved PARP. Lipid accumulation and effect on the differentiation of 3T3-L1 cells (adipocytes) were tested with date seed extract treatments. The influence of date seed polyphenols on glucose uptake was studied in 3T3-L1 cells and C2C12 cells (muscle cells). The antioxidant activity of the polyphenols from date seed products such as date seed extract (DSE), date seed powder (DSP), and date seeds fortified bread (DSB) was tested following *in-vitro* digestion to study their stability in the gastrointestinal milieu. DSE treatment resulted in significantly reduced viability in MCF-7 and Hep-G2 cells with 48-h treatments. Glucose uptake increased in the adipocytes with DSE treatments; moreover, it inhibited adipocyte differentiation and lipid accumulation. DSE decreased the expression levels of PPAR-γ, C/EBPα, adiponectin and upregulated GLUT-4, and phospho-AMPK. This study also found that date seed samples retained antioxidant activity in the digestive milieu and concludes that the date seed polyphenols remain active in the digestive milieu and exhibit potential anti-hyperglycemic and anti-adipogenic activity.

## Introduction

Due to multiple health properties, polyphenols receive increasing attention as a potential nutritional therapeutic agent against several chronic diseases. Numerous *in-vitro* studies provide evidence on a range of bioactivity of polyphenols and polyphenol-rich extracts in affecting platelet functions (1), exerting cardioprotective activity (2), preventing systemic inflammation by acting as immunomodulatory and anti-inflammatory agents (3), as anti-tumour agents by inducing apoptosis and blocking cell proliferation (4, 5), exerting anti-obesity (6, 7), and anti-diabetic effect (8–10). *In-vivo*, the health benefit of polyphenols is primarily attributed to their antioxidant effect. By quenching free radicals, they can protect cell constituents, including DNA, against oxidative damages (3).

Date (*Phoenix dactylifera L*.) seeds (DS) are a rich source of polyphenols and an industrial by-product with minimal human use (11–13). The polyphenol content of DS ranges between 1864.82 and 4768.87 mg gallic acid equivalent/100 g (13). The polyphenol profile of DS revealed flavan-3-ols, especially catechins and epicatechins, as the most abundant group, with values ranging from 47.91 to 50.18 g/kg flavan-3-ols (12, 14). Apart from flavan-3-ols, DS also contain significant quantities of phenolic acids as protocatechuic acid, p-hydroxybenzoic acid, and caffeoylshikimic acid (11, 12, 14). Consequently, DS are a promising source of bioactive compounds. Therefore, they could play a role in the prevention and/or treatment of chronic diseases. This assertion is supported by previous *in-vivo* studies in both animal and human. The dietary supplementation of date seed extract (DSE) in rats resulted in a significant decrease in the tissue oxidation levels and a subsequent strengthening of antioxidant status (15–17). A human study demonstrated an increase in antioxidant activity even with a single dose of date seed products such as DSE, date seed powder (DSP), and date seed supplemented Arabic pita bread (DSB) (18). Moreover, many polyphenols such as catechin, epicatechin, p-hydroxybenzoic acid, protocatechuic acid, and procyanidins have reported anti-tumour property (19–24). Although these compounds are abundant in DS, there is no detailed investigation of the anti-tumour potential of DS. An earlier study by Habib et al. explored the anti-proliferative activity of DSE in pancreatic cancer cells (25). Besides, the polyphenol profile of DS is also in favour of possible anti-hyperglycemic and anti-adipogenic effect. Studies have suggested that protocatechuic acid and hydroxybenzoic acid can alter cellular signalling by binding to a specific receptor, enhancing the antioxidant mechanism in the cell (26). These findings were clearly demonstrated by protocatechuic acid's ability to activate the antioxidant response element (ARE) through Nrf-2 signalling (27). Indeed, protocatechuic acid exhibits insulin-like activity by activating peroxisome proliferator-activated receptor-gamma (PPAR-γ), a nuclear hormone receptor that controls glucose and lipid metabolism (28). It can also affect other critical regulators of adipogenesis like CCAAT enhancer-binding protein (C/EBP) and adipokines (29, 30). Besides, catechin, procyanidins, and protocatechuic acid were shown to suppress adipocyte differentiation *in-vitro* and *in-vivo* studies (29–31). Nevertheless, no data is available about the constitutive effect of these polyphenols, which are abundantly present in DSE. Bioavailability is a critical element that governs the bioactivity of natural dietary compounds. The recent study of the *in-vitro* bioaccessibility of DS polyphenols highlighted the quick release and absorption of phenolic acids such as protocatechuic acid and caffeoylshikimic acid (14). However, the change in antioxidant activity of the DS polyphenols during the *in-vitro* digestion, was not quantified. These findings will be crucial to understand the effectiveness of using DS as a polyphenol source in human.

The present study aims to shed light on the influence of *in-vitro* digestion on the DSE, DSP, DSB to quantify the changes happening to date seed polyphenols in the stomach and small intestine. Besides, due to the abundance of bioactive polyphenols such as protocatechuic acid, hydroxycinnamic acids, procyanidins, catechins, and epicatechins in DS, we hypothesise potential *in-vitro* biological effects of DSE including anti-proliferative effect against cancer cell lines, potential modulation of hyperglycemia and anti-adipogenic property. The present study investigates the anti-tumour potential of DS by testing its effectiveness in inducing apoptosis in a panel of cancer cell lines, the anti-hyperglycemic activity of DSE in preadipocyte 3T3-L1 cells and muscle cells C2C12 and the anti-adipogenic effect in preadipocyte 3T3-L1 cells.

## Materials and Methods

### Materials

All chemicals and enzymes used in the study were purchased from Sigma-Aldrich (St. Louis, MO, USA) unless stated otherwise. For the cell culture experiments, the various cancer cell lines MCF-7, MDA-MB-231, Caco2, PC-3 and HepG2, and mouse 3T3-L1 fibroblast, C2C12 myoblasts were obtained from the European Collection of Authenticated Cell Cultures (ECACC) (Salisbury, UK). Trypsin, rosiglitazone, 3-isobutyl-1-methylxanthine (IBMX), human recombinant insulin (4 mg/mL), and Oil Red O stain were also purchased from Sigma Aldrich (St. Louis, MO, USA). Cell viability assay kit and 2-[N-(7-Nitrobenz-2-oxa-1,3- diazol-4-yl)amino]-2-deoxy-d-glucose (2-NBDG) was obtained from Abcam (Cambridge, UK). Dulbecco's Modified Eagles Medium (DMEM) (Gibco, 12800-017), foetal bovine serum (FBS) (Gibco, 10437-028), horse serum (Gibco, 16050-122), phosphate-buffered saline (Gibco, 10010-023), penicillin-streptomycin cocktail (Gibco, 15140-148), and 0.25% Trypsin (Gibco, 25200-056) were obtained from Thermo-Fisher Scientific (Waltham, MA, USA). Date seeds from the Khalas variety were supplied by Al Foah Company, Al Ain, UAE. Date seed powder (DSP) and date seed extract (DSE) were prepared as described by Hilary et al. (14). Date seed supplemented Arabic pita bread (DSB) was baked in-house using the procedure as described by Platat et al. (32).

### Anti-tumour Activity of DSE

#### Cell Lines

Five cancer cells lines were used in the study; human breast adenocarcinoma cell lines MCF-7 and MDA-MB-231, human colon adenocarcinoma cell line Caco-2, human hepatocyte carcinoma cell line HepG2, and human prostate adenocarcinoma cell line PC-3. All cell lines were cultured in Dulbecco's Modified Eagles Medium (DMEM) supplemented with 10% foetal bovine serum (FBS), 2 mM glutamine, 1% non-essential amino acids, 100 U/mL penicillin and 100 μg/mL streptomycin.

#### Cell Viability Assay

Cell viability in the five cancer cell lines was evaluated with tetrazolium salt, WST-1 (4-[3-(4-Idophenyl)-2-(4-nitro-phenyl)-2H-5-tetrazolio]-1,3-benzene) assay. For the experiment, 0.01 × 10^6^ cells were seeded in 96 well-plates in their respective culture medium and maintained for a day in a 5% CO_2_ incubator at 37°C. After 24 h, the culture medium containing date seed extract in the range of concentrations between 50 and 3,000 μg/ml was introduced to the cells. The cells were incubated with extract for 24, 48, and 72 h, following 10 μl of WST-1 was added in each well including blank (without cells) and control (untreated) and incubated for 5–6 h in a 5% CO_2_ incubator at 37°C. After incubation, the absorbance of the samples at 480 nm was measured using a spectrophotometer (Multiscan Go, Thermo-Fisher Scientific, MA, USA). The data was calculated against control groups as percentage viability.

#### PARP Cleavage Assay

PathScan^®^ Cleaved PARP (Asp214) Sandwich ELISA Kit (Cell Signalling Technology, MA, USA) was used to measure the cleaved PARP levels in the cancer cell lines after treatment with varying concentration of date seed extract. The concentrations of date seed extract and the treatment duration were standardised based on the viability assay results. Briefly, 0.3 × 10^6^ cells were seeded on six-well plates, and the cells were maintained for 12 h in a 5% CO_2_ incubator at 37°C before introducing culture medium with date seed extract in the range of concentrations 10–1,000 μg/ml. Following incubation for 48 h, the cells were harvested, and cleaved PARP was measured following the manufacturer's protocol.

### Anti-adipogenic and Anti-hyperglycemic Activities of DSE

#### Differentiation of 3T3-L1 and C2C12 Cells

For differentiation, 3T3-L1 preadipocytes were cultured in proliferation media (DMEM containing 10% FBS and 100 U/mL penicillin and 100 μg/mL streptomycin) for 2 days. After the cells attained 100% confluency, the culture was continued for two more days in the same media. After 2 days (cell stage 0), the cells were treated with differentiation media (DMEM containing 10% FBS, 0.5 mM IBMX, 1 μM dexamethasone, and 1 μg/ml insulin) for 3 days to induce differentiation, following which the cells were exposed to maturation media (DMEM containing 10% FBS and 1 μg/mL insulin) for another 2 days. Finally, the maturation media was replaced with proliferation media and the culture was continued until day 8 with regular media change. For differentiation of C2C12 myoblast, cells were grown to 90% confluency in DMEM supplemented with 2% horse serum. The media was changed every 2 days, and culture the culture was maintained for 8 days. The formation of the C2C12 myotube confirmed the differentiation of the cells.

#### Oil Red O Staining

3T3L1 cells were differentiated in the presence of varying concentrations of date seed extract (10–100 μg/ml) to study the effect of DSE on adipogenesis. The toxicity of DSE was evaluated with cell viability assay to standardise the concentrations for the experiment. The cells were stained with Oil Red O stain after differentiation. Briefly, the cells were washed with PBS to remove dead cells and treated with 10% formaldehyde for 5 min to remove any water remnants. Following this, the cells were fixed in 10% formaldehyde for 1 h and then washed with 60% isopropanol for 1 min. The cells were then dried, and oil red O stain was introduced to the cells and incubated for 10 min. After 10 min of incubation, the cells were washed with distilled water four times, and the lipids in cells were eluted using 100% isopropanol. The absorbance of the sample was measured at 500 nm using a spectrophotometer (Multiscan Go, Thermo-Fisher Scientific, MA, USA).

#### Glucose Uptake Assay

The assay was performed using Glucose Uptake Assay Kit (ab136955; Abcam) by following the manufacturer's protocol. In brief, the differentiated C2C12 and 3T3-L1 cells were starved in serum-free DMEM/F12 medium overnight, followed by 40 min of incubation in Krebs–Ringer–Phosphate–Hepes buffer. Subsequently, cells were treated with 50, 100, and 200 μg/ml of DSE for 2 h; 10 mM-2-deoxyglucose was added for an additional 20 min. Next, cells were washed three times with cold PBS and lysed with extraction buffer, frozen at −80°C for 10 min and heated at 85°C for 40 min. After cooling on ice for 5 min, the lysates were neutralised by adding neutralisation buffer and centrifuged. The remaining lysate was then diluted with assay buffer. Finally, the colourimetric product generation was set up by two amplification steps according to the manufacturer protocol. Absorbance was measured at 412 nm using a spectrophotometer (Multiscan Go, Thermo-Fisher Scientific, MA, USA).

#### Western Blotting

Date seed extract-treated 3T3-L1 cells were harvested using a cell scraper into a fresh tube and washed with phosphate buffer saline (PBS). Two hundred microliter of RIPA cell lysis buffer (50 mM Tris-HCl pH 7.4, 150 mM NaCl, 1 mM ethylenediaminetetraacetic acid, 1% Triton X100, 0.1% sodium dodecyl sulphate (SDS), 10 mM NaF, 1 mM Na_3_VO_4_, 50 mM Na_4_P_2_O_7_) containing 1% protease inhibitor cocktail, 1 mM phenyl methyl sulfonyl fluoride (PMSF), and 10 mM dithiothreitol was added to the cells. After lysis, the protein content of cell lysate were determined to adjust uniform sample loading. The samples were treated in sample buffer (0.1 M Tris-HCl pH 6.8, 2% SDS, 12% β-mercaptoethanol, 20% glycerol, and 0.2% bromophenol blue) by boiling at 95°C for 5 min. Proteins were separated using 12% SDS-PAGE. Separated proteins were electrotransferred into a nitrocellulose membrane. The membranes were blocked with 5% non-fat milk in tris buffered saline containing 0.1% Tween-20 (TBS-T) for 1 h and then incubated with primary antibodies overnight. The secondary antibody was introduced to blot for 2 h after washing the blot with TBS-T four times. The blot was finally developed by chemiluminescence using a Chemi Doc imaging system (Bio-Rad Laboratories, USA). The following antibodies were used: Primary antibodies- anti-adiponectin, anti-PPARγ, anti-C/EBPα, anti-glut4, anti-phospho-AMPK, anti-AMPK (Cell Signalling Technology, MA, USA), and anti-β-actin (Santa Cruz Biotechnology, TX, USA); secondary antibodies- anti-rabbit IgG and anti-mouse IgG (Jackson Immune Research, Cambridge House, UK).

### *In-vitro* Digestion of DS

The date seed samples were digested following the protocol described by Hilary et al. (14). Briefly, 1 g of DSP, 1 g of DSB, and 500 mg of DSE were mixed with 10 mL of saline (140 mM NaCl, 5 mM KCl, 150 μM BHT). The samples were further diluted with the saline solution to 18 ml. The solution was then acidified to pH 2 with 1 M HCl. Next, 0.5 mL of pepsin solution (0.2 g of pepsin in 5 ml of 0.1 M HCl) was added and incubated in a shaking water bath at 37°C for 1 h for gastric digestion. The pH of the sample was then increased to 6.9 with 1 M NaHCO_3_ and 2.5 ml of pancreatin-bile solution (0.45 g of bile extract and 0.075 g of pancreatin in 37.5 ml of 0.1 M NaHCO_3_) was added to the samples. Finally, the samples were incubated in a shaking water bath at 37°C for 2 h and centrifuged at 7,000 rpm for 15 min, and the supernatant was retrieved and stored at −80°C for further analysis.

### Total Polyphenol Assay

Folin's assay was used to determine the total phenolic content (33). Briefly, 100 μl of date seed samples (digested and undigested DSB, DSE or DSP) or gallic acid standard was mixed with 200 μl of Folin–Ciocalteu reagent and 800 μl of sodium carbonate solution were added to the mixture within 30 s to 8 min of addition of Folin-Ciocalteu reagent. The samples were incubated at room temperature for 120 min under dark conditions, and absorbance was measured at 750 nm using a spectrophotometer (Multiscan Go, Thermo-Fisher Scientific, MA, USA). The total phenolic content of the sample was expressed as mg of gallic acid equivalents (GAE) per gramme of the sample.

### Antioxidant Activity Assays

#### DPPH Assay

DPPH radical scavenging capacity of digested and undigested date seed samples was determined using the method described by Janaszewska and Bartosz (34). First, 20 μl of the sample or standard trolox was added to 380 μl of 10 mM sodium phosphate buffer (pH 7.4), and 400 μl of 0.1 mM DPPH in methanol was added to this mixture. Then, the mixture was incubated for 30 min at room temperature under dark conditions, and absorbance was read at 517 nm using a spectrophotometer (Multiscan-Go, Thermo-Fisher Scientific, MA, USA). The results were calculated against the standard curve build from trolox, and the antioxidant activity was expressed as trolox equivalents (TE).

#### FRAP Assay

Ferric Reducing Antioxidant Power (FRAP) assay was performed to study the reducing power of the undigested and digested samples of DSB, DSE and DSP following the method of Benzie and Strain (35). First, 20 μl of the sample or standard trolox was added to 600 μl of freshly prepared working FRAP reagent [300 mM acetate buffer (pH 3.6), 10 mM TPTZ in 40 mM HCl and 20 mM FeCl_6_ in the ratio of 10:1:1, pre-warmed to 37°C]. Then, the mixture was incubated for 5 min, following which the absorbance of the mixture was read at 593 nm using a spectrophotometer (Multiscan Go, Thermo-Fisher Scientific, MA, USA). The results were calculated against the standard curve build from trolox, and the antioxidant activity was expressed as TE.

#### ABTS Assay

Trolox Equivalent Antioxidant Activity (TEAC) was measured using scavenging activity of the digested and undigested DSP, DSE, and DSB samples against ABTS using the method described by Re et al. (36) with minor modifications. First, 600 μl of ABTS solution in 10 mM sodium phosphate buffer (pH 7.4, initial absorbance at 734 nm of 0.7) was added to 10 μl or standard trolox. After 5 s, the mixture's absorbance was measured at 734 nm using a spectrophotometer (Multiscan Go, Thermo-Fisher Scientific, MA, USA). The results were calculated against the standard curve build from trolox scavenging activity.

### Statistical Analysis

Statistical analysis for the experiments was performed using GraphPad Prism software version 8.1.2. All experiments were performed in triplicates. The Shapiro-Wilk test and Kolmogorov-Smirnov test checked model assumptions for the data for normality. ANOVA with multiple comparisons was performed for all quantitative parameters to identify the test groups' treatment effect. A *p*-value < 0.05 was considered statistically significant. The same software was used to build the graphs in the whole study.

## Results

### Cell Viability and PARP Cleavage

**Figure 1:**
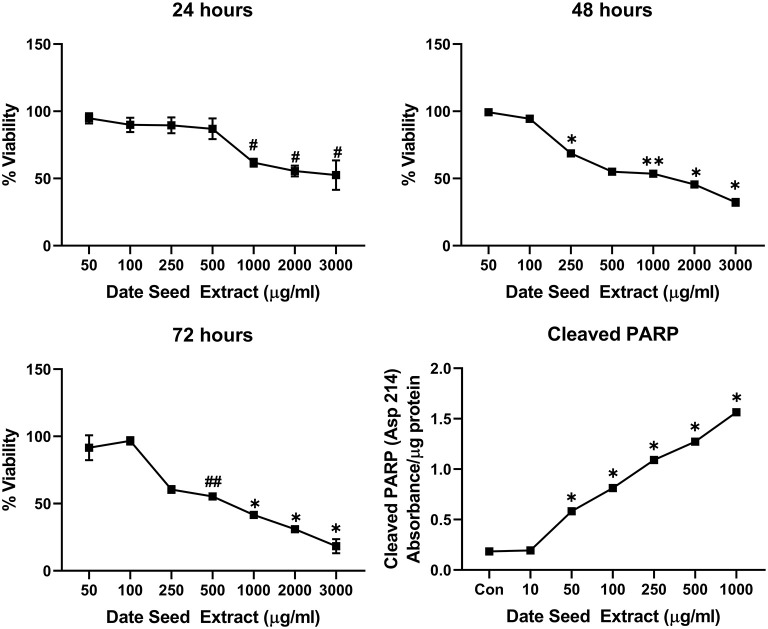
Cell viability and cleaved PARP assay in MCF-7 cells. Data presented as mean ± s.d. ANOVA with multiple comparisons were performed, and statistical significance set at *p* ≤ 0.05. #Significant compared to 50, 100, 250, and 500 μg/ml date seed extract, ^*^significant compared all treatment groups, ^**^significant compared to all treatment groups except 500 μg/ml date seed extract, ##significant compared to all treatment groups except 250 μg/ml date seed extract.

**Figure 2:**
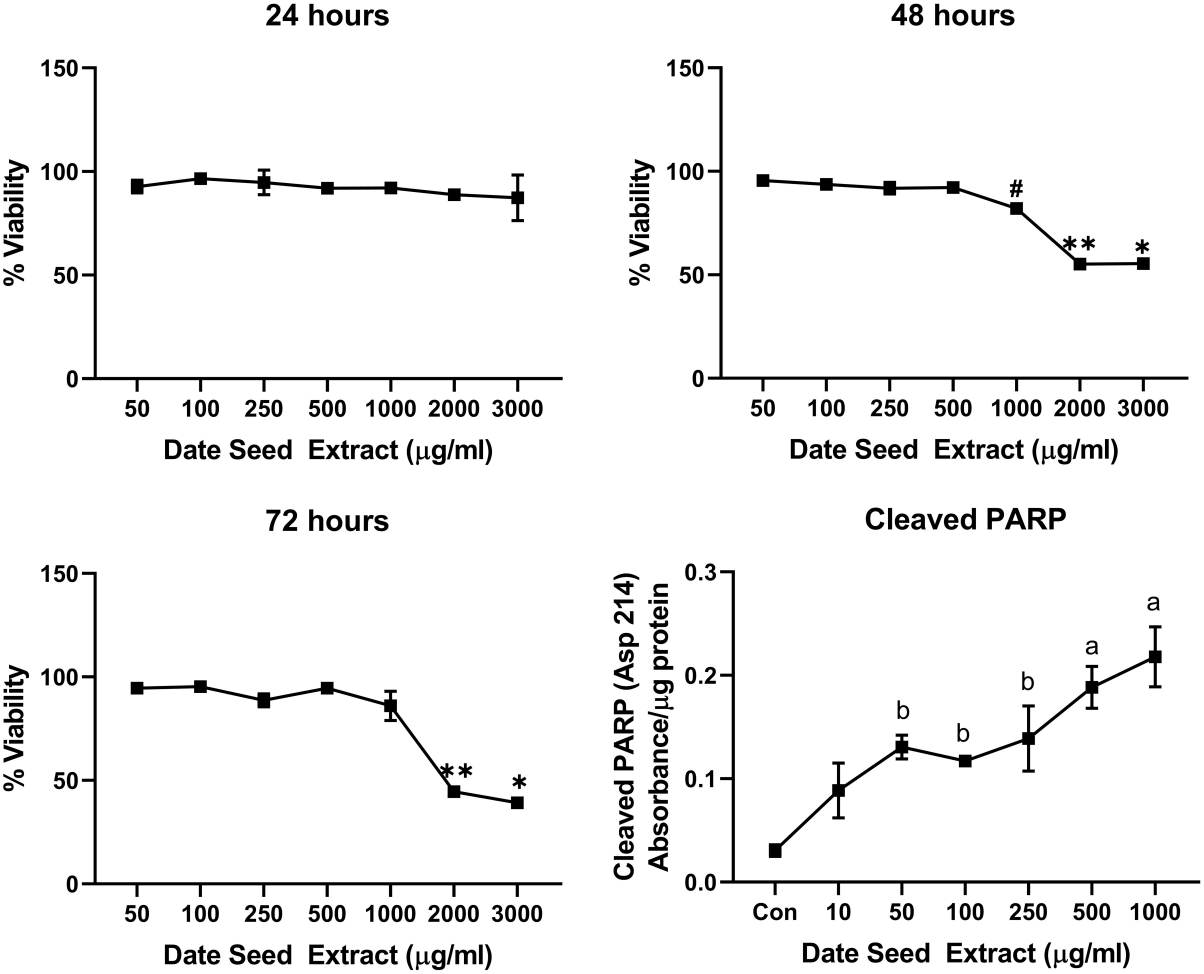
Cell viability and cleaved PARP assay in MDA-MB-231 cells. Data presented as mean ± s.d. ANOVA with multiple comparisons were performed, and statistical significance set at *p* ≤ 0.05. ^*^Significant compared to all treatment groups except 2,000 μg/ml, ^**^significant compared to all treatment groups except 3,000 μg/ml, #significant compared to 50, 100, 250, and 500 μg/ml, a significant compared to control, 10, 50, 100, and 250 μg/ml date seed extract, b significant compared to control and 10 μg/ml date seed extract.

**Figure 3:**
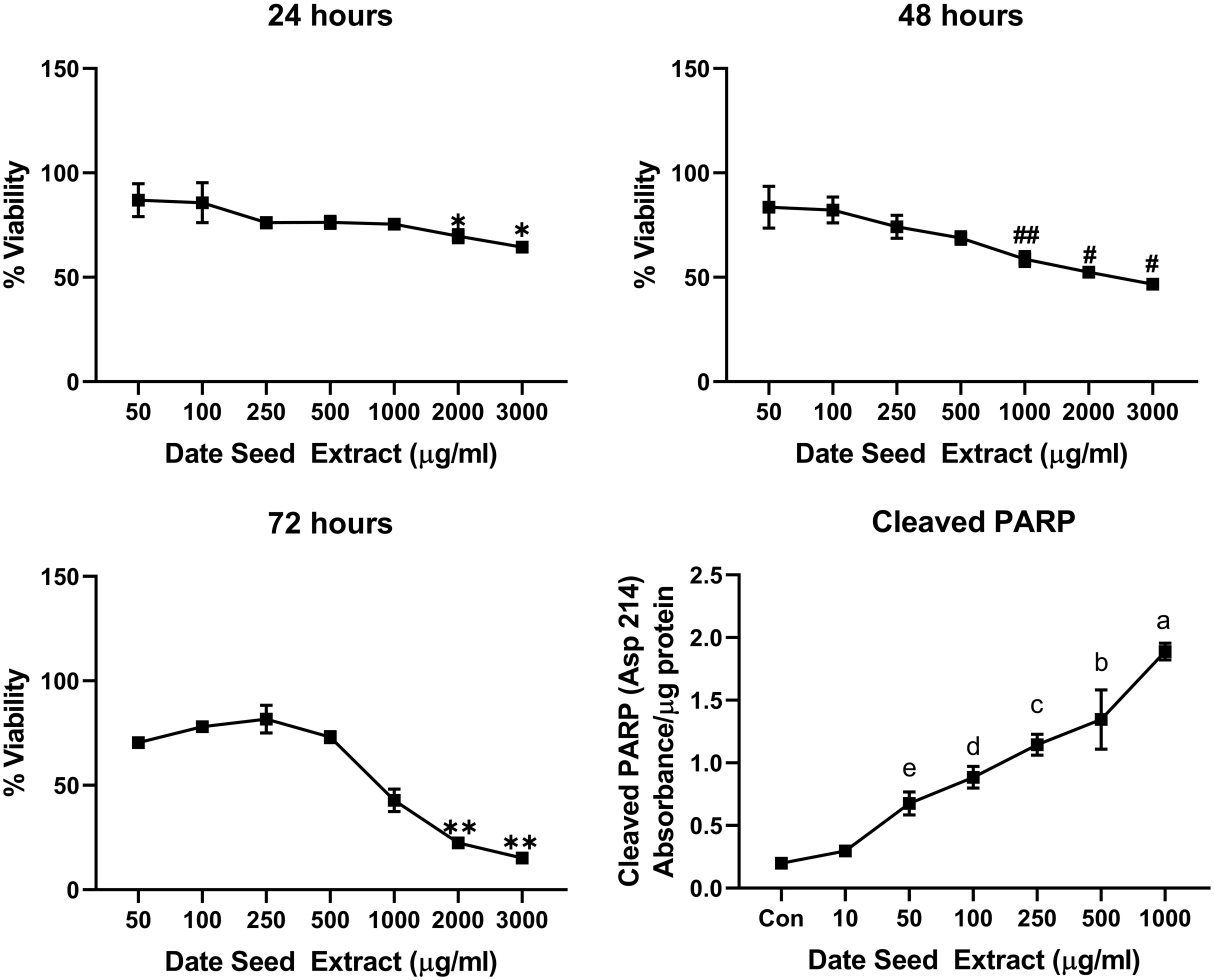
Cell viability and cleaved PARP assay in Caco-2 cells. Data presented as mean ± s.d. ANOVA with multiple comparisons were performed, and statistical significance set at *p* ≤ 0.05. ^*^Significant compared to 50 and 100 μg/ml date seed extract, #significant compared to 50, 100, 250, and 500 μg/ml date seed extract, ##significant compared to 50, 100, and 250 μg/ml date seed extract, ^**^significant compared to all other treatment groups, a significant compared to all treatment groups, b significant compared to all treatment groups except 250 μg/ml, c significant compared to all treatment groups except 100 and 500 μg/ml, d significant compared to all treatment groups except 250 and 50 μg/ml, e significant compared to control and 10 μg/ml.

**Figure 4:**
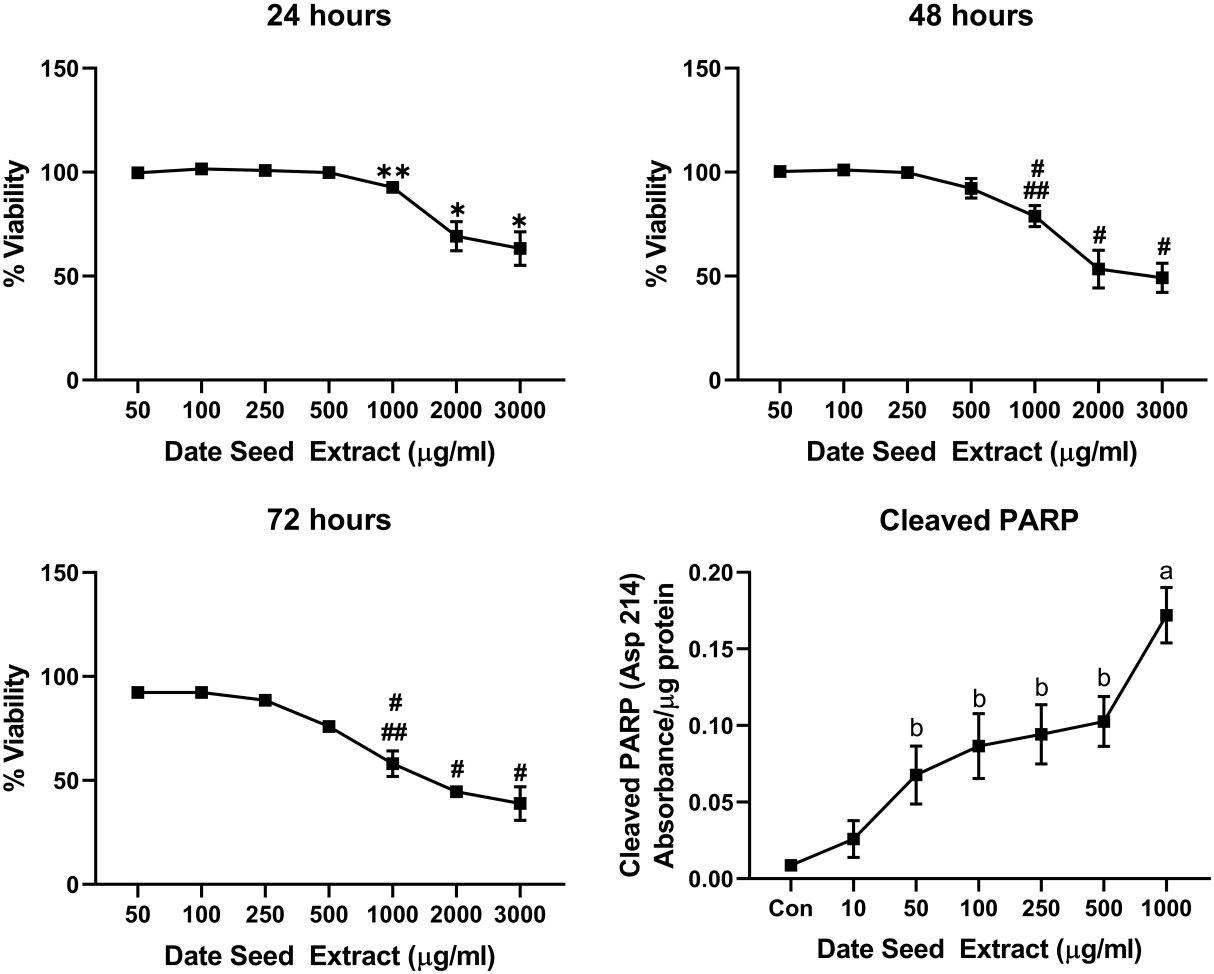
Cell viability and cleaved PARP assay in PC-3 cells. Data presented as mean ± s.d. ANOVA with multiple comparisons were performed, and statistical significance set at *p* ≤ 0.05. ^*^Statistically significant compared to 50, 100, 250, 500, and 1,000 μg/ml date seed extract, ^**^significant compared 100, 250 μg/ml date seed extract, #significant compared to 50, 100, 250, and 500 μg/ml date seed extract. ##significant compared to 2,000 and 3,000 μg/ml date seed extract, a significant compared to all treatment groups, b significant compared to control and 10 μg/ml date seed extract.

**Figure 5:**
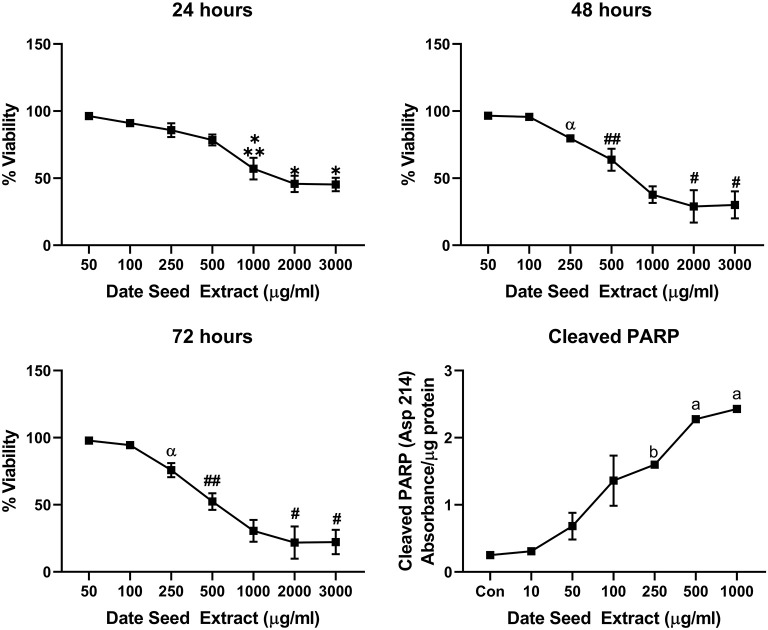
Cell viability and cleaved PARP assay in HepG2 cells. Data presented as mean ± s.d. ANOVA with multiple comparisons were performed, and statistical significance set at *p* ≤ 0.05. ^*^Statistically significant compared to 50, 100, 250, and 500 μg/ml date seed extract, ^**^significant compared to 2,000 and 3,000 μg/ml date seed extract, #significant compared to 50, 100, 250, 500, and 1,000 μg/ml date seed extract, ##significant compared to 50, 100, 250 μg/ml date seed extract, α significant compared to 100 and 50 μg/ml date seed extract, a significant compared to all treatment groups, b significant compared to all treatment groups except 100 μg/ml date seed extract.

Five cancer cell lines, MCF-7, MDA-MB-231, Caco2, PC-3, and HepG2, were treated with DSE to study its cytotoxic effect. The cells were exposed to a concentration ranging from 50 to 3,000 μg/ml DSE for three different periods, 24, 48, and 72 h. In MCF-7 cells (Figure 1), a significant decrease in viability was observed with a higher DSE concentration (1,000, 2,000, and 3,000 μg/ml). The trend continued with higher incubation periods of 48 and 72 h. After 48 h, the cells' viability significantly dropped with a lower 250 μg/ml concentration. The amount of cleaved PARP also increased with decreasing viability in a dose-dependent manner after 48 h of incubation. In the case of MDA-MB-231 (Figure 2), no significant change in viability was observed with DSE treatment after 24 h, but after 48 h, a significant decrease in viability was observed from the concentration 1,000 μg/ml. Similar results were obtained after 72 h of incubation, from the concentration of 2,000 μg/ml. The cleaved PARP levels were increasing significantly in the cells in a dose-dependent manner even at lower concentrations after 48 h. In Caco-2 cells (Figure 3), cell viability was reduced significantly with the two highest DSE concentrations (2,000 and 3,000 μg/ml), after 24 and 72 h incubation and from 1,000 μg/ml, after 48 h. Cleaved PARP analysis also revealed a dose-dependent increase starting with a lower concentration of 50 μg/ml DSE. In the PC-3 cell line (Figure 4), treatment reduced cells' viability after 24 h with DSE concentrations ≥1,000 μg/ml. The effect was similar to increasing treatment times of 48 and 72 h. The PARP data is in agreement with the viability results. There was an increasing trend of cleaved PARP starting with DSE doses of 50 μg/ml, but it did not significantly reduce viability. However, we observed the highest cleaved PARP content in the cells with a DSE dose of 1,000 μg/ml. In the HepG2 cell line (Figure 5), viability reduced after 24 h with DSE concentrations concentration ≥1,000 μg/ml similar to PC-3. With increasing incubation times of 48 and 72 h, we observe that 500 μg/ml DSE was also influential in lowering viability. The observation was confirmed with increasing levels of cleaved PARP levels in the cells DSE treatment. The IC-50 doses for DSE treatments were calculated from the viability data for all cell lines, but the values were < 1,000 μg/ml only in MCF-7 and HepG2 cell lines. The IC-50 dose in MCF-7 was 678.4 μg/ml DSE, followed by HepG2 cells with 662.2 μg/ml, both with an exposure time of 48 h.

### Anti-adipogenic Activity of DSE

**Figure 6:**
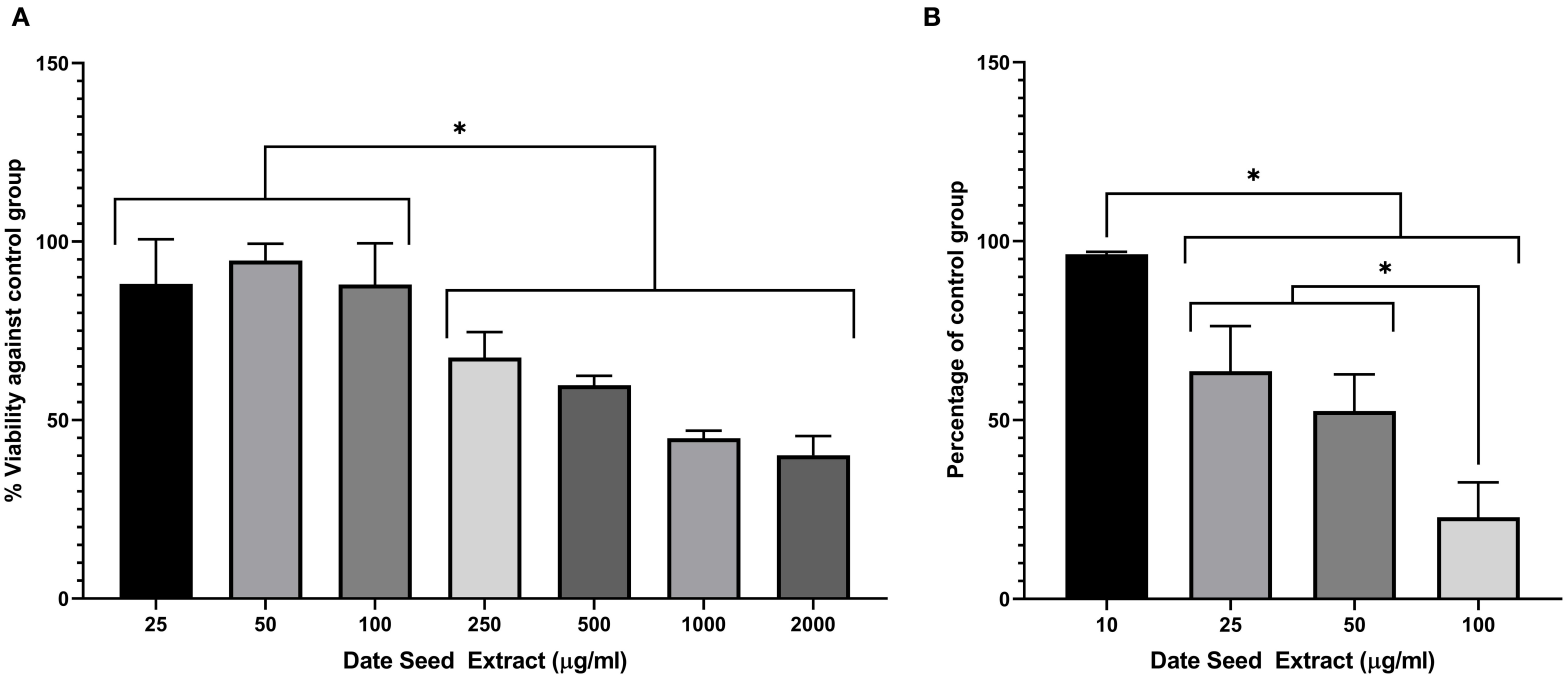
**(A)** Viability assay in 3T3-L1 cells after treatment with date seed extract. **(B)** Lipid accumulation assay in 3T3-L1 cells. Data expressed as a percentage calculated against untreated control for both **(A,B)**. Mean ± s.d presented. ANOVA with multiple comparisons was performed, and statistical significance set at *p* ≤ 0.05, ^*^significant difference between treatment groups.

**Figure 7:**
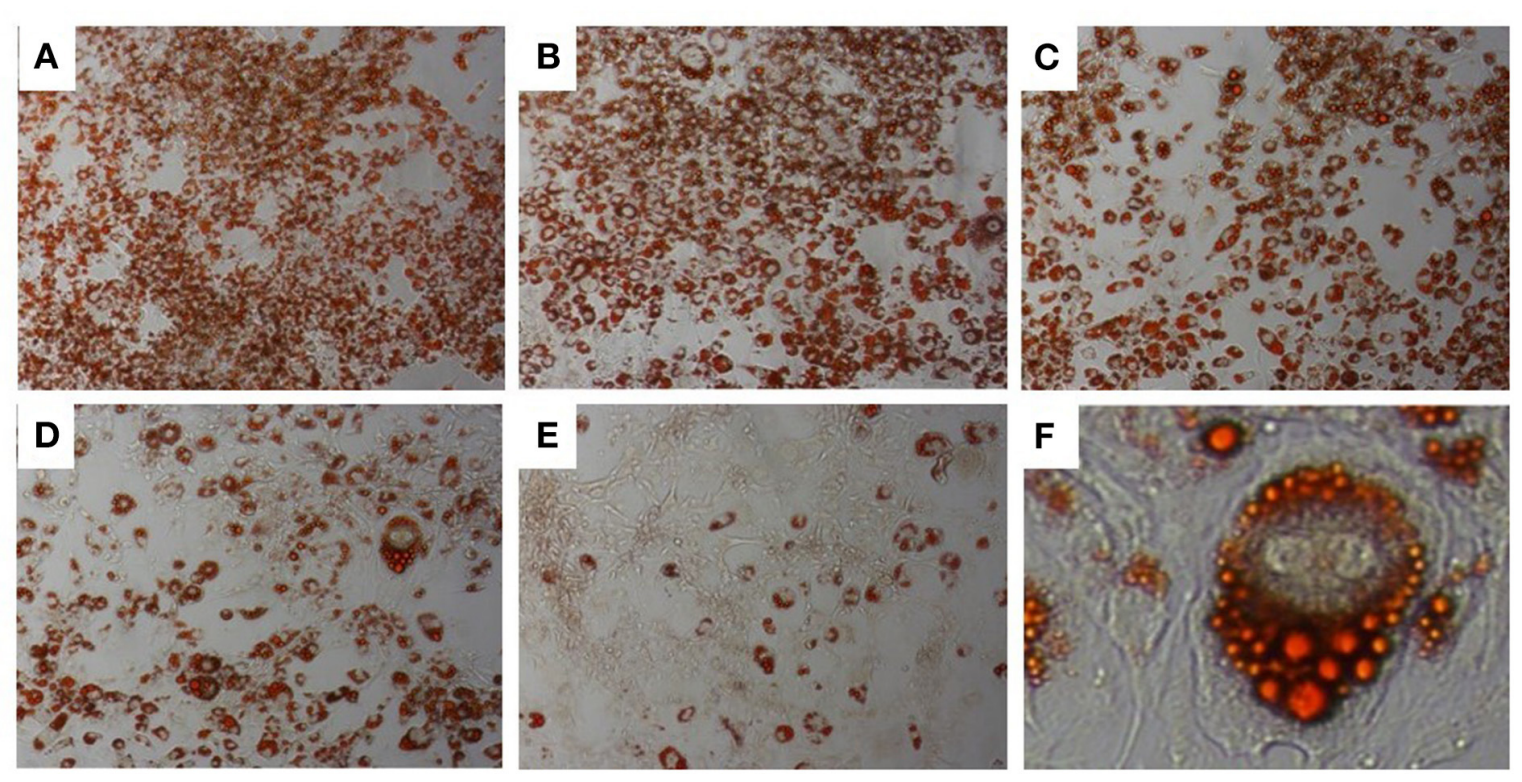
Oil Red-O Staining in 3T3-L1 cells. 3T3-L1 adipocytes differentiated from mouse 3T3-L1 fibroblasts with DSE concentrations **(A–E)** 0, 10, 25, 50, and 100 μg/ml, respectively. Magnification **(A–E)** 20X. **(F)**-40X magnification.

A range of DSE concentrations (10–100 μg/ml) was added to the differentiation media, and lipid accumulation was studied to investigate the effect of DSE on the differentiation of 3T3-L1 cells to mature adipocyte. The DSE concentration for the experiment was standardised by cell viability analysis of DSE on 3T3-L1 cells (Figure 6A). We observed a significant decrease in the cells' viability with a concentration higher than 100 μg/ml. Hence, for the adipogenesis inhibition experiment, the maximum concentration of 100 μg/ml was chosen to rule out the possibility of cytotoxicity of DSE. Microscopic evaluation of the cells following Oil Red O staining confirmed the presence of oil droplets accumulated in the differentiated cells (Figure 7). This analysis highlighted the decrease of oil droplets in the differentiated cells with increasing extract concentration (Figure 6B). The lowest fat droplets were observed with the highest concentration studied (100 μg/ml). The inhibitory effect of DSE on triglyceride accumulation is dose-dependent, and 10 μg/ml of DSE did not have any significant effect, and we observed a notable decrease in lipid content from 25 to 100 μg/ml DSE.

### Glucose Uptake in 3T3L1 and C2C12 Cells

**Figure 8:**
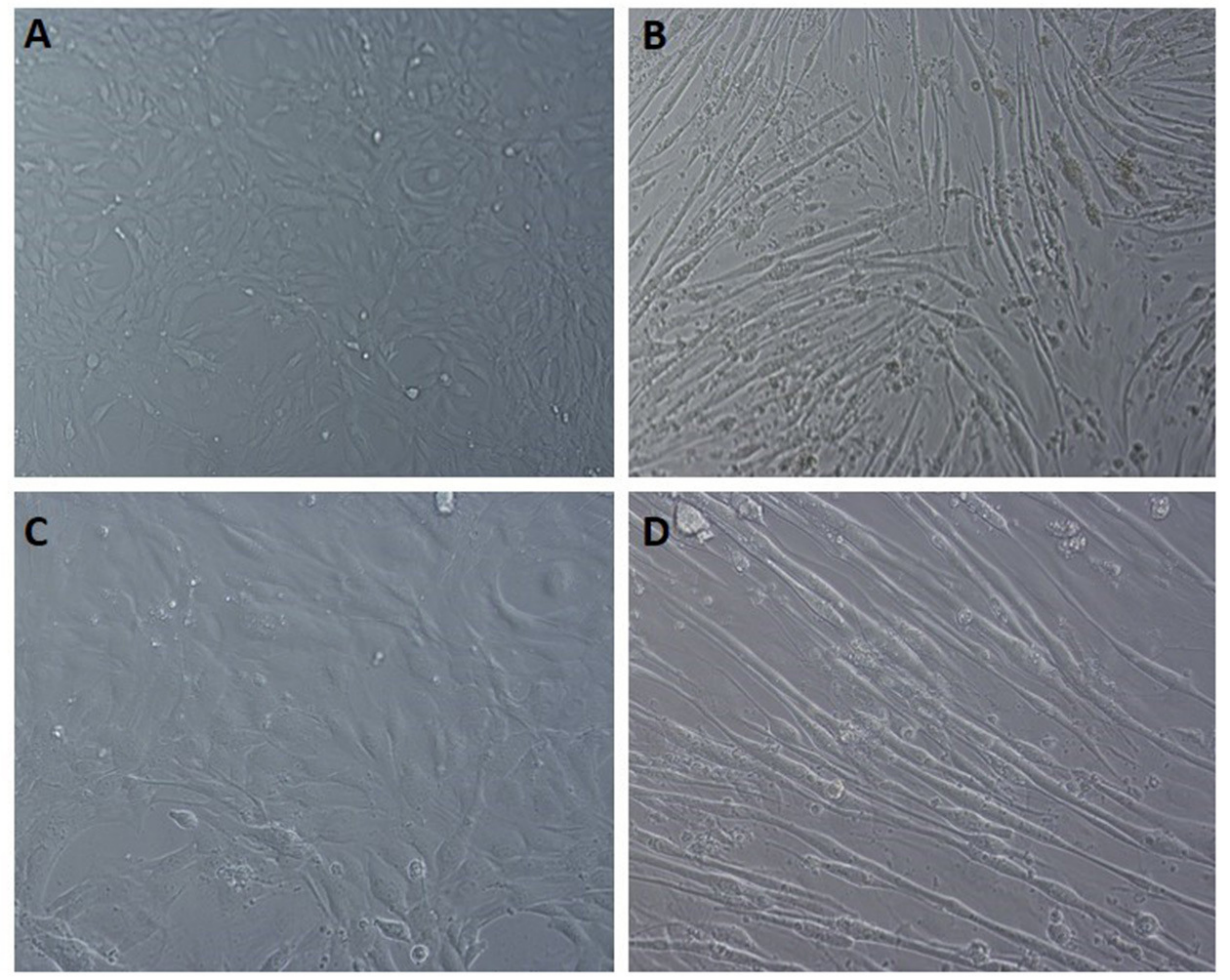
Light microscopic images of C2C12 differentiation. **(A,C)** undifferentiated cells at 10x field and 20x magnification, respectively. **(B,D)** differentiated cells displaying the myotube formation at 10x and 20x magnification, respectively.

**Figure 9:**
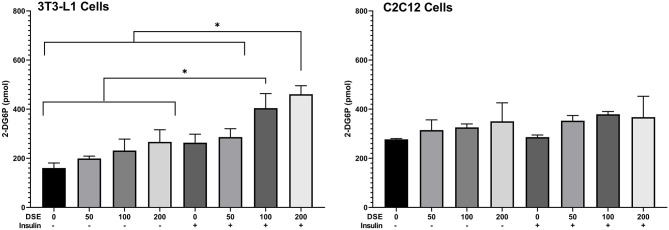
Glucose uptake assay in 3T3-L1 and C2C12 cells. Data expressed in pmol 2-D6P detected. DSE, date seed extract (μg/ml). (+) insulin supplemented group, (–) insulin-deficient group. Mean ± s.d presented. ANOVA with multiple comparisons was performed, and statistical significance set at *p* ≤ 0.05, ^*^significant difference between treatment groups.

Glucose uptake assay was performed in the presence and absence of insulin to understand the metabolic changes in the adipocyte and muscle cells with DSE treatment. Fully differentiated 3T3L1 cells (Figure 8) were exposed to DSE 1 h before the addition of glucose-6-phosphate. Glucose-6-phosphate uptake and the enzymatic reaction gives the proportional colour variation based on the extent of uptake. As shown in Figure 9, three concentration of 50, 100, and 200 μg/ml were used in the study. In the case of 3T3-L1 cells, DSE induced glucose uptake in a dose-dependent manner. In the absence of insulin, we observed an increase in glucose uptake with increasing concentration, but the observed differences were not significant. However, a significant increase in glucose uptake was detected with 200 μg/ml of DSE+insulin than insulin supplemented control in 3T3L1. Nevertheless, the increase in glucose uptake with 100 μg/ml DSE + insulin was significant only compared to the insulin-deficient test group, which would only indicate the effect of insulin. Hence, a synergistic effect of DSE was observed with 200 μg/ml in 3T3-L1 cells. Glucose uptake was generally not improved in the C2C12 cells with DSE treatments. The results in C2C12 cells were inconsistent due to the high variability in the replicates.

### DSE Downregulates the Expression of Adipogenesis Specific and Related Proteins

**Figure 10:**
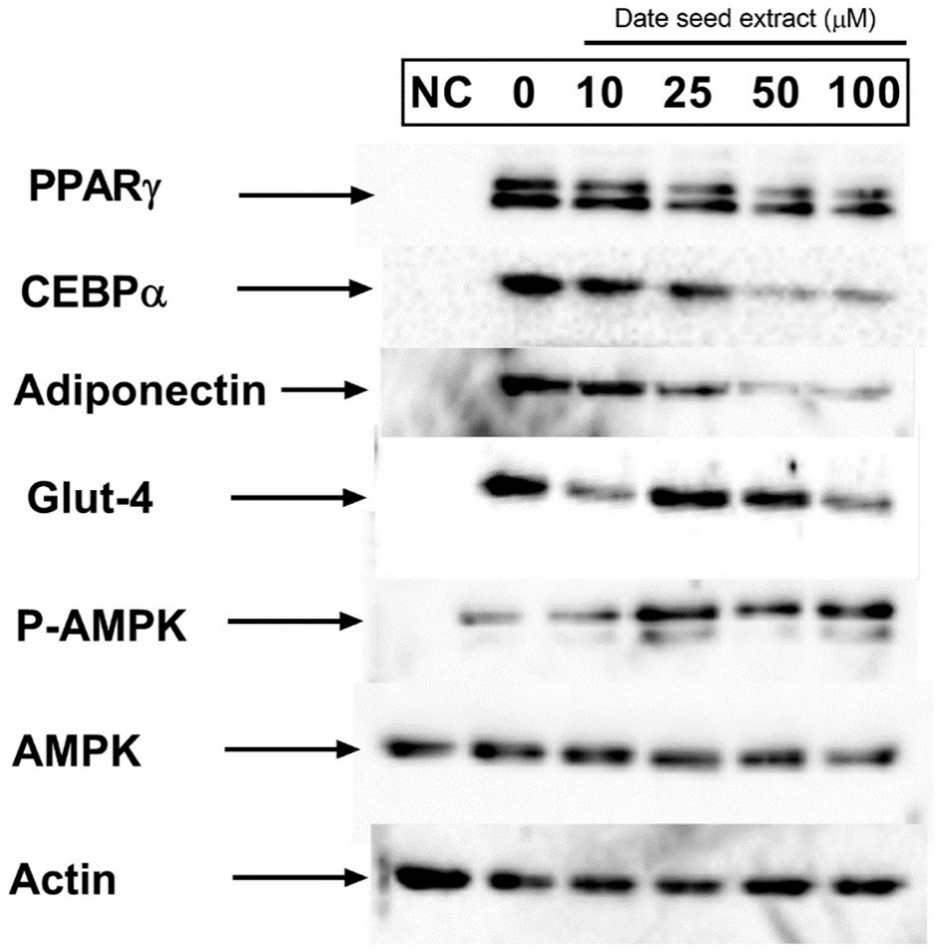
Effect of date seed extract on the critical adipogenic transcription factors; PPAR γ and C/EBP-α, adiponectin, GLUT-4, p-AMPK, and AMPK. 3T3-L1 adipocytes were incubated with or without DSE concentrations (0–100 μ) for 24 h. NC, Untreated control.

The expression of adipogenic transcription factors PPARγ and C/EBPα are considered as the critical regulator of adipocyte differentiation. Western blot analysis (Figure 10) revealed the downregulation of PPARγ and C/EBPα expression with increasing DSE concentration. Another critical adipogenic regulator, adiponectin, was also downregulated with DSE in a dose-dependent manner. Besides, glucose transporter 4 (GLUT4), an important biomarker in adipogenesis, is also elevated significantly in response to DSE treatment. These results suggest that polyphenols of DSE inhibited lipogenesis and adipogenesis by downregulation of adipogenic marker proteins. The AMPK activation with DSE was evaluated to investigate the mechanism behind the adipogenesis. Adipocyte differentiation has predominantly increased the AMPK phosphorylation in the treatment group as compared to the control group. Treatments with DSE at 10, 25, 50, and 100 μg/mL has enhanced the phosphorylation of AMPK in a dose-dependent manner.

### Polyphenol Content During *in-vitro* Digestion

**Figure 11:**
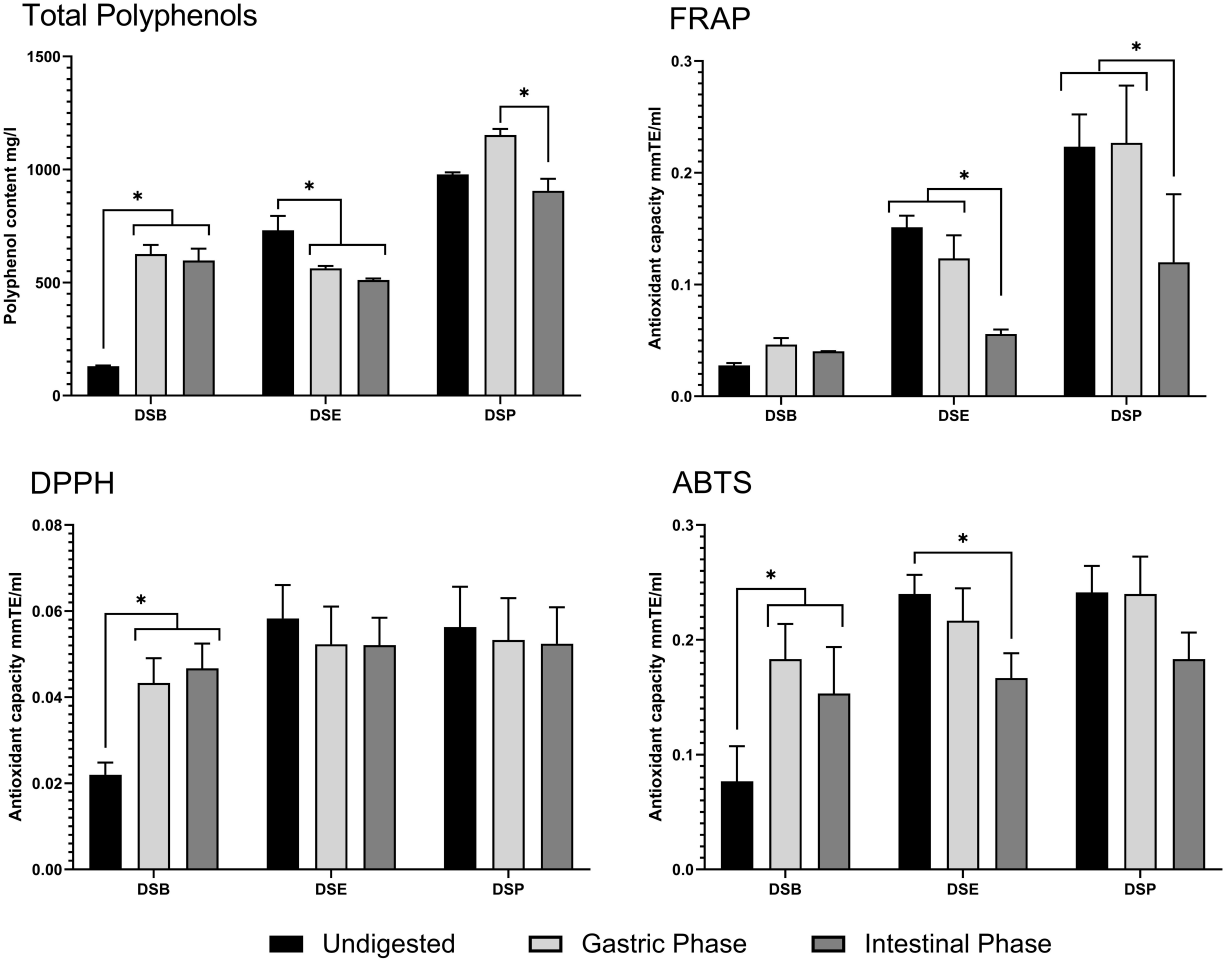
Total polyphenol content and antioxidant activity of date seed sample during *in-vitro* digestion. Data presented as mean ± s.d. ANOVA with multiple comparisons were performed, and statistical significance set at *p* ≤ 0.05, ^*^significant difference. DSB, date seed bread; DSE, date seed extract; DSP, date seed powder.

The total polyphenol content of DSP, DSE and DSB was tested in undigested food material after the gastric phase and intestinal phase of *in-vitro* digestion. The results of the experiment are provided in Figure 11. In DSB, a higher polyphenols content after the gastric phase, compared to the content of undigested DSB, was observed and is maintained even after the intestinal phase of digestion. Similarly, in DSP, there is an apparent increase in the content of polyphenols following the gastric phase of digestion; however, the level was significantly reduced compared to the gastric phase after intestinal digestion and was similar to the original level in undigested DSP. The polyphenolic content of DSE was lowered after the gastric phase digestion and was remained the same after intestinal digestion.

### Antioxidant Activity During *in-vitro* Digestion

The antioxidant capacity of DSP, DSE, and DSB were tested during the various stages of *in-vitro* digestion to assess any possible change in the bioactivity due to the digestion process. DPPH radical scavenging assay (Figure 11) indicates a significant increase in the trolox equivalent antioxidant capacity after gastric digestion of DSB, compared to undigested DSB. This increase was maintained after intestinal digestion. In this assay, no changes were observed in the antioxidant capacity of DSE and DSP levels compared to the undigested samples. Results from the FRAP assay were different (Figure 11). No variations were observed in DSB samples during digestion. Simultaneously, there was a successive significant decrease in the antioxidant power as the digestion progressed from gastric to intestinal phase in DSP and DSE, compared to undigested samples. The antioxidant power after intestinal digestion was significantly lower compared to both undigested food materials and the samples from the gastric phase in the case of both powder and extract. The results of the TEAC assay using ABTS reagent are similar to the DPPH assay in DSB and DSP (Figure 11). A significant decrease was observed in DSE after the intestinal digestion compared to the undigested sample.

## Discussion

Due to their reported antioxidant properties, polyphenols offer potential nutritional strategies in combating several chronic diseases such as diabetes, cancer, cardiovascular diseases, osteoporosis, and even neurodegenerative diseases (37, 38). We observe the evidence to this claim from epidemiological data that demonstrates that consuming polyphenol-rich diets is associated with a reduced risk of developing chronic diseases. Its bioaccessibility and bioavailability govern the effectiveness of polyphenols ingested. The chemical changes occurring in the digestive milieu is a critical factor that affects the antioxidant activity of polyphenols *in vivo*. Date seeds are an ideal source of polyphenols owing to their polyphenol richness. If antioxidant effects of date seeds have already been demonstrated in both animals and humans, the digestive process's impact on the native polyphenols from DS and the resulting bioactivity remains unknown. Besides, the other health properties usually associated with polyphenols, including anti-tumour, anti-adipogenic, and anti-hyperglycemic effect, remain to be explored with date seeds.

Date seeds are rich in flavan-3-ols (catechin and epicatechin, polymeric proanthocyanidins) and phenolic acids such as protocatechuic acid caffeoylshikimic acids. The polyphenol profile indicates a potential anti-tumour activity. Earlier, Habib et al. (25) reported the anti-tumour potential of DSE in the pancreatic cells. In the current study, we investigated the potential in five cancer cell lines. We found that DSE effectively reduced viability only at high concentrations of 1,000 μg/ml or above with 24-h treatment. The observation was confirmed in all cell lines except MDA-MB-231, where no significant cell death was observed. Our observations indicate that DSE was ineffective in reducing viability in cancer cells unless a very high concentration of 1,000 μg/ml were exposed to the cells for a longer duration. The only data indicative of anti-tumour effect were observed in MCF-7 and HepG2, where after 48 h, DSE treatments with a lower concentration of 250 μg/ml reduced viability. Earlier, Al-Zubaidy et al. had demonstrated the anti-proliferative effect of date seed polyphenols extracted with chloroform in MCF-7 cells (39). A recent study in MDA-MB-231 cells with DSE found a significant decrease in viability with higher concentrations (>1,000 μg/ml), which is also not effective enough to claim anti-tumour activity (40). MDA-MB-231 is a more aggressive late-stage model of breast cancer, which would explain the difference in activity observed between MCF-7 and MDA-MB-231. Contrary to our results, a previous study with protocatechuic acid from black rice bran has indicated that purified protocatechuic acid can induce apoptosis in cells even with range of low concentrations such as 10–50 μg/ml (20). Similarly, numerous studies have reported the effect of procyanidin B type dimer and trimers to be effective against cancer cells (22, 23, 41). Nevertheless, in our present study, despite the presence of these polyphenols in DSE, it failed to be effective in concentrations < 1,000 μg/ml. This could be attributed to the relative abundance of highly polymeric proanthocyanidins in DSE which does not cross the plasma membrane. These compounds could be masking the effect of the comparatively lower concentrations of other bioactive polyphenols such as protocatechuic acid, procyanidins, and caffeoyl shikimic acids, which are known to pass the plasma membrane. Based on our data, a constitutive effect of phenolic acids on the cancer cells lines cannot be attributed to DSE, as they may not reach the target cells in quantities significant enough to exert toxicity. Nevertheless, the observed cells death in our experiment were due to the induction of apoptosis. The apoptotic pathway induction was observed with PARP cleavage after 48-h treatment with DSE. Cleaved PARP increased in a dose-dependent manner in all cell lines, confirming apoptosis in cells. PARP cleavage is a typical hallmark of caspase-dependent apoptosis (42).

A synergistic improvement in glucose uptake was observed when treating 3T3-L1 cells with insulin in combination with DSE. Protocatechuic acid is reported to stimulate the insulin-dependent pathway by mimicking insulin action (43). Previous reports showed that epicatechin, epigallocatechin and non-gallate-type catechins also enhanced glucose uptake in 3T3-L1 cells in a dose-dependent manner (44). Therefore, DSE, in which these compounds have been reported and are known to cross the intestinal barrier (14), could be responsible for improving glucose uptake. The increase in glucose uptake with DSE treatment in insulin demonstrates enhancement of the insulin signalling pathway by date seed polyphenols. Additionally, catechin can regulate glucose metabolism in muscle cells (45). A derivative of catechin and epigallocatechin from the tea promoted the glucose uptake in the skeletal muscle cells (46). Moreover, procyanidin dimers and trimers also enable glucose uptake by aiding translocation of GLUT-4 through AMPK-dependant pathway in skeletal muscle (47). We did observe an improved glucose uptake with DSE treatment in C2C12 muscle cells; however, the results were not significant. The degree of polymerisation of procyanidin compounds in DSE can highlight the differences observed in our study between the two cell lines. In skeletal muscle cells, it is reported that hyperglycemic activity of procyanidins is different with differences in the degree of polymerisation. A high degree of polymerisation favours hypoglycemia through inhibition of α-glucosidase activity in small intestine and lower degree of polymerisation helps translocation of GLUT-4 via AMPK-pathway (47). Hence, the relative abundance of high polymers in DSE could impede the activity of procyanidin dimers and trimers in DSE (14) to bring about a significant change in glucose uptake in C2C12 cells by GLUT-4 translocation. It could be inferred that any potential anti-hyperglycemic effect of DSE polyphenols could be attributed to its ability to inhibit α-glucosidase during digestion.

Our result also demonstrates the effectiveness of DSE in inhibiting adipocyte differentiation. PPAR-γ, adiponectin and C/EBP-α are well-recognised as adipogenic transcription factors (48). Adipogenic inhibition by polyphenols can be controlled by inhibiting the signalling pathway of PPAR-γ, adiponectin and C/EBP-α proteins in the cells. PPAR-γ is a transcription factor that has been connected to regulating adiponectin gene expression in adipose tissue (49). We observed downregulation of both PPAR-γ and adiponectin in 3T3-L1 cells with DSE treatments. DSE also altered the expression level of C/EBP-α, an upstream regulator of PPAR-γ. Adiponectin is a crucial protein produced entirely in the adipocyte during the differentiation process—an increase in adiponectin results in increased lipid accumulation in adipocytes. Adiponectin regulates metabolic activity by regulating insulin sensitivity or fatty acid stimulation in tissues. The liberation of adiponectin is controlled with DSE to diminish adipocyte differentiation. Thus, the result throws light on the mechanism involved in reduced lipid accumulation in 3T3-L1 cells observed with Oil Red O staining. Our results are in agreement with a recent study by Etesami et al. in 3T3-L1 cells (50). This study reported decreased differentiation potential in 3T3-L1 and downregulated expression of C/EBP-α and PPAR-γ with DSE treatment. However, it is crucial to note that the DSE used by Etesami et al. is an aqueous extract prepared with heat. The lowest effective concentration in the study was 315 μg/ml. Our results demonstrate that date seed polyphenols extracted with ethanol: water (1:1) was effective at a concentration as low as 25 μg/ml. Such differences are expected as most polyphenols in date seeds such as hydroxybenzoic acids, hydroxycinnamic acids, and flavan-3-ols are sensitive to heat. AMPK is a serine/threonine-protein kinase that plays a significant role in regulating glucose, lipids, and cholesterol metabolism. The data presented here showed that polyphenols from DSE effectively inhibited lipid accumulation during the 3T3-L1 differentiation process by suppressing adipocyte-specific proteins and activating the phosphorylation of AMPK in all concentration levels. Since AMPK activation inhibits cell proliferation, it consecutively attenuates the expression of the adiponectin, PPAR-γ, and C/EBPα (51). The results when converged also highlights the molecular mechanism involved in the increased glucose uptake in 3T3-L1 cells with DSE treatment.

The present study investigated the total polyphenol content and antioxidant activity during simulated *in-vitro* digestion to quantify the change occurring during digestion. Three different forms of date seed proposed for human use was investigated; DSP, DSE, and DSB. Total polyphenol content after digestion increases in DSB, indicating their release from the food matrix during the digestive process. During digestion, macromolecules get favourable pH condition for the hydrolysis and polyphenols that are bound to it are released (52, 53). Therefore, increased polyphenol content promote antioxidant properties in the digested samples of date seed bread. This assertion is possible because polyphenols are responsible for the antioxidant activity of the samples; therefore, the increase in the total phenolic content increases the antioxidant activity correspondingly (54). From the results, both DPPH radical scavenging assay and TEAC assay, the antioxidant power of DSB following digestion was improved significantly, indicating the release of polyphenol from the food matrix. However, a decrease in the polyphenol content was observed in DSE, which led to differing antioxidant activity levels between the three assays in the study.

As demonstrated from the DPPH radical scavenging assay, the antioxidant activity remained unchanged despite the decrease in total polyphenol content. At the same time, FRAP and TEAC recorded a successive decrease in the antioxidant activity at the end of intestinal digestion. The results in DSE are not surprising since it is a crude mixture of polyphenols that are being subjected to the digestive process. Polyphenols are known to undergo irreversible structural changes by auto-oxidation, isomerisation and conjugation in alkaline pH during intestinal digestion, but some species are more stable than others dependent on the structure (55, 56). Such dynamics are expected to change the polyphenolic content and, thereby, antioxidant activity. DSE is a crude mix of the free polyphenolic fraction, is more likely affected by the digestive process. In DSP, the total polyphenol content increases at the gastric stage due to polyphenols' release from the food matrix. However, the levels are significantly reduced in the intestinal phase due to the digestive process's effect. A similar observation was made by Srisena et al. (57) in their study where subsequent lowering of date seed polyphenols such as procyanidin B, catechins, and epicatechin was observed from the gastric phase to the intestinal phase as the digestion progressed. A solid food matrix such as DSP has to be disrupted and the polyphenols solubilised into digestive fluids to be bioaccessible. Most polyphenols are located in the plant cell vacuole and apoplast, where they are usually conjugated to monosaccharides, polysaccharides, and proteins (58). Once they are released from the cell upon cell wall breakdown due to processing or metabolism, free polyphenols may form interactive associations with proteins and dietary fibre in the food matrix (59). Losses of polyphenols between gastric and intestinal digestion stages have been observed before; for example, Correa-Betanzo et al. (60) reported a loss of 44% in wild blueberry.

## Conclusions

The study concludes that DSE does not exert significant cytoxicity in MDA-MB-231, Caco-2, and PC-3 cell lines at clinically relevant doses (< 1,000 μg/ml). The cytotoxic potential of DSE in cancer cell lines was limited to MCF-7 and Hep G2 cells with a treatment period of 48 h in our study. However, the polyphenols in DSE can inhibit lipid accumulation and differentiation of 3T3-L1 cells. The anti-adipogenic potential of DSE is achieved by down-regulation of key adipogenic regulators such as adiponectin, PPAR-γ, and C/EBPα. Consequently, there is upregulation of phosphorylated AMPK in 3T3-L1 cells and upregulation of GLUT-4, resulting in increased glucose uptake by the cells, pointing to a possible anti-hyperglycemiceffect. The present study also demonstrates that date seeds polyphenols, when ingested either as a powder or extract or when fortified in food such as date seed bread, retain their antioxidant activity in the digestive milieu, highlighting the significance of date seed polyphenols as a suitable dietary strategy to combat chronic conditions such as obesity and diabetes. Although a range of polyphenols in the crude mixture such as procyanidin, protocatechuic acids or catechins could be suspected to bring about the effect, further studies must elucidate which of these polyphenols are responsible for the changes to the specific molecular markers.

## Data Availability Statement

The original contributions presented in the study are included in the article/supplementary material, further inquiries can be directed to the corresponding author.

## Author Contributions

CP and WI designed the project and obtained the research funding. CP also participated in data analysis, reviewing, and finalising the manuscript. SH performed the experiments in collaboration with JK, US, and FA-M. SH conducted the statistical analysis and drafted the manuscript. All authors have read and approved the final manuscript.

## Conflict of Interest

The authors declare that the research was conducted in the absence of any commercial or financial relationships that could be construed as a potential conflict of interest.

## Publisher's Note

All claims expressed in this article are solely those of the authors and do not necessarily represent those of their affiliated organizations, or those of the publisher, the editors and the reviewers. Any product that may be evaluated in this article, or claim that may be made by its manufacturer, is not guaranteed or endorsed by the publisher.
